# Supplementary material for: The Dually Localized EF-Hand Domain-Containing Protein TgEFP1 Regulates the Lytic Cycle of Toxoplasma gondii
Source: Cells. 2022 May 21;11(10):1709. doi: 10.3390/cells11101709 (PMC9139715; doi:10.3390/cells11101709)
Supplement: Supplementary file 1 [file cells-11-01709-s001.zip › cells-1733461-supplementary.pdf]

Supplemental materials

Table S1. Primers used in this study.

Purpose	Name	Sequence
Generate vector to tag endogenous TgEFP1	EFP1HA forward	cccccgcgcttcctgccaccaagcttcgccaggctgt
	EFP1HA reverse	accgttctcgccagtttcacCAtCctgcaaGtgcatagaaggaa
Generate pSag1-Cas9-U6-sg1EFP1 by using Q5 mutagenesis	EFP1SG1 forward	ccaacggagagttttagagctagaaatagc
	EFP1SG1 reverse	agtcgaactcaacttgacatccccatttac
Amplifying donor template for knockout clone 1	DonorKO1 forward	ggacgaagacgacgaggccttcatccgcgaggaattcatggaacaaaagttgatttctgaagaag
	DonorKO1 reverse	tgcttctttcctcctctgtgttcctgctttttgacgcctagcggaagatccgatcttgc
Generate pSag1-Cas9-U6-sg2EFP1 by using Q5 mutagenesis	EFP1SG2 forward	ttccggctaggttttagagctagaaatagc
	EFP1SG2 reverse	gaaagaaaacaacttgacatccccatttac
Amplifying donor template for knockout clone 1	DonorKO2 forward	aggtccgagtgacgcatcccgaaatcggaaacagcgaactgaacaaaagttgatttctgaagaag
	DonorKO2 reverse	cgatattctgcgagagtcaagagtccatcttggttggagtgcggaagatccgatcttg
Amplifying TgEFP1 gDNA to make complementing construct	TgEFP1comp WT forward	ggaggacgggaattcaaggagatggggacggcg
	TgEFP1comp WT reverse	gttccctagggaattcaatagctgatgatacatactcgcgatatt
Deleting signal peptide from complementing construct	ΔSP forward	ccaccatgttcgcgcgcctggcgtcg
	ΔSP reverse	gcgcgaacatggtggcaggaagcgc
Generation of D97A complementation construct using Q5	D97A forward	catggagtacgcatccaacggag
	D97A reverse	aattcctcgcggatgaag
Generation of D129A complementation construct using Q5	D129A forward	ccggctagtggcatccaaccaag
	D129A reverse	aagaaagaaaacagttcgc

Targeting complementing template to Ku80 locus	KU80 forward	gtccccggttcgcctcagcacacacacacatgacgtacatcgaggga acaaaagctgggtac
	KU80 reverse	ggatagctccattgtttctgatgggaactattccgacattacattcatcctg caagtgcatagaaggaa

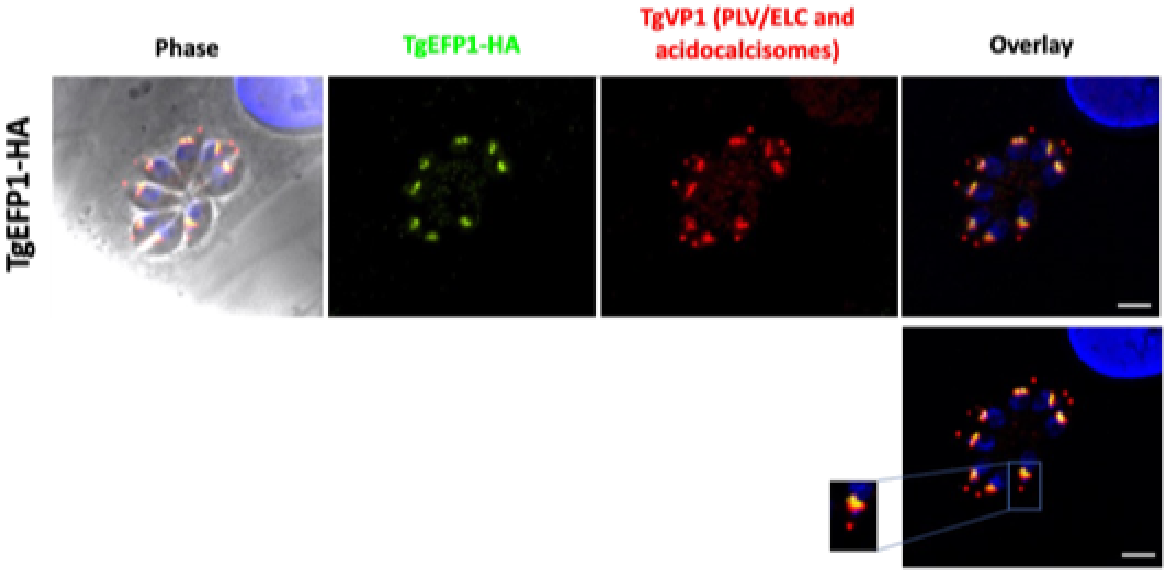

Figure S1: TgEFP1 co-localizes with TgVP1 only at the PLV/ELC and not at the acidocalcisome. Intracellular parasites stained for TgEFP1-HA (green) and TgVP1 (red). Overlay shows co-localization (yellow) of TgEFP1 and TgVP1 at the PLV/ELC. TgVP1 localization is also observed at the acidocalcisomes which is apically localized (red). Scale bars represent 2μM.
